# Biomarkers of Oxidative Stress and Endogenous Antioxidants for Patients with Chronic Subjective Dizziness

**DOI:** 10.1038/s41598-020-58218-w

**Published:** 2020-01-30

**Authors:** Zhigang Fang, Keer Huang, Chang-Hyun Gil, Jin-Woo Jeong, Ho-Ryong Yoo, Hyeong-Geug Kim

**Affiliations:** 1 Guangzhou University of Chinese Medicine, Guangzhou, 510405 PR China; 2 The First Affiliated Hospital of Guangzhou University of Chinese Medicine, Guangzhou, 510405 PR China; 3 Department of Biochemistry and Molecular Biology, Indiana University School of Medicine, Indianapolis, IN 46202 USA; 4 Division of Vascular Surgery, Department of Surgery, Indiana University School of Medicine, Indianapois, IN 46202 USA; 5 Researcher, Animal & Plant Utilization Team, Nakdonggang National Institute of Biological Resources, 137, Donam 2-gil, Sangju-si, Gyeongsangbuk-do 37242 Korea; 6 Department of Neurology Disorders, Dunsan Oriental Hospital of Daejeon University, 75, Daedeok-daero 176, Seo-gu, Daejeon, 35253 South Korea

**Keywords:** Cytokines, Predictive markers

## Abstract

As a neurotologic disorder of persistent non-vertiginous dizziness, chronic subjective dizziness (CSD) arises unsteadily by psychological and physiological imbalance. The CSD is hypersensitivity reaction due to exposure to complex motions visual stimuli. However, the pathophysiological features and mechanism of the CSD still remains unclearly. The present study was purposed to establish possible endogenous contributors of the CSD using serum samples from patients with the CSD. A total 199 participants were gathered and divided into two groups; healthy (n = 152, male for 61, and female for 91) and CSD (n = 47, male for 5, female for 42), respectively. Oxidative stress parameters such as, hydrogen peroxide and reactive substances were significantly elevated (*p* < 0.01 or *p* < 0.001), whereas endogenous antioxidant components including total glutathione contents, and activities of catalase and superoxide dismutase were significantly deteriorated in the CSD group (*p* < 0.01 or *p* < 0.001) as comparing to the healthy group, respectively. Serum levels of tumor necrosis factor -α and interferon-γ were significantly increased in the CSD participants (*p* < 0.001). Additionally, emotional stress related hormones including cortisol, adrenaline, and serotonin were abnormally observed in the serum levels of the CSD group (*p* < 0.01 or *p* < 0.001). Our results confirmed that oxidative stress and antioxidants are a critical contributor of pathophysiology of the CSD, and that is first explored to establish features of redox system in the CSD subjects compared to a healthy population.

## Introduction

Since mid of two thousand years, chronic subjective dizziness (CSD) has been used as termination of medial field to explain the specific pathological conditions of persistent, non-vertiginous dizziness, subjective imbalance, and hypersensitivity to motion cues in the lack of active vestibular deficits^1,2^. The above symptoms of the CSD are often worsened with stimulation of visual environments or settings with indistinct visual orientation cues.

Etiologically the CSD is mainly arisen from neurotologic or other pathological events including acute vertigo, unsteadiness, or dizziness, and they are corresponded to vestibular neuritis, pre-syncope, or panic attacks^3^. Some studies also revealed that approximately 25% of patients with the CSD experienced the above events, however underlying pathophysiological mechanisms are needed to reveal obscurely^4–6^.

The main and general symptoms of the CSD are represented by emotional behaviors, therapeutic accesses are also very limited. Only some of treating methods are available including vestibular therapy^7^, cognitive behavioral therapy^8,9^, or selective serotonin reuptake inhibitors (SSRI) which was generally used to treat depression^1,2,10^, which is focusing on the relieving the main symptoms.

Thus, to comprehend the exact pathophysiological condition of the CSD, take apart from relying on the symptoms, we aimed to observe to find out a convincing contributor to accomplish the CSD in serum levels which are mainly focusing on the endogenous redox system as comparing with healthy populations.

## Material and Methods

### Ethic approval

The present study was completed in Daejeon Dunsan Oriental Hospital of Daejeon University after approval of the Institutional Review Board (IRB) of Daejeon Oriental Medical Centre (authorization number: DJOMC-34-Ver. 1.00). This IRB was approved by Ministry of Food and Drug Safety in South Korea. It was performed by accordance with the Helsinki Declaration of 1975 (as sixth revision in 2008) and the Guidelines for Good Clinical Practice approved from Korea FDA since Aug. 2005 (Approval No. 110). From May, 1^s^t, 2009 to December, 31^st^, 2015, and a total 199 subjects were informed about the study via a standardized leaflet and provided written consent.

### Study design

Present study enrolled via answering of questionnaire surveys and divided into two groups (aged 19 to 65 years) that were composed of healthy adults with no symptoms about physical and psychological distress. A physician and radiologist totally examined potential candidates at Dunsan Oriental Hospital of Daejeon University (Daejeon, South Korea). Populations who were dropped out the present study were excluded subjects as follows; any hematological or radiological test abnormalities, night workers, alcohol drinkers, smokers, took any kinds of medication or health supplements, severely obese or lean (body mass index, BMI > 30 or <17), respectively. Participants who were met the criteria of CSD were followed by previous studies which were slightly modified for adapting in the current study^11–13^; *(1) Persistent (over than 3 months) sensation of non-vertiginous dizziness that may include one or more of the following vague descriptors likewise ‘lightheadedness’, ‘heavy headedness’, ‘a feeling of imbalance that frequently is not apparent to others, ‘a feeling that the “inside of their head’ is spinning in the absence of any perception of movement of the visual surround’, ‘a feeling that the floor is moving from underneath them’, ‘a feeling of disassociation from one’s environment’, (2) Chronic hypersensitivity to one’s own motion or the movement of objects in the environment, (3) Exacerbation of symptoms in settings with complex visual stimuli, such as grocery stores or shopping malls, or when performing precision visual tasks, for instance workers with a computer*, respectively.

### Questionnaire surveys of dizziness handicap index (DHI) and visual analogue scale (VAS)

The questionnaire survey was performed by DHI, which consists a total 25 items for assessing self-perceived disability associated with dizziness in 3 areas (physical, functional, and emotional)^14^. Additionally, all participants were asked to indicate their feeling of general dizziness by drawing a vertical line on a 10 cm.

### Blood collection, laboratory test, and biochemical analyses

Whole blood of all participants were isolated from forearm needle and Vacutainer™ tubes by a trained phlebotomist. After collection of blood in tubes containing ethylenediaminetetraacetic acid (EDTA) was incubate at RT for removal fibrinogen then centrifugation at 3,000 × *g* for 20 minutes at 4 °C. Then, all serum samples were immediately transferred to micro tubes for multiple aliquots at −70 °C until analyzed. In the current study, to avoid circadian rhythm which may affect to the stress hormone secretion, we collected serum samples after fasting conditions of 12 hours in the 2:00–3:00 pm from arm vein through all participants.

Hematology tests for red blood cell (RBC) and its related parameters including hemoglobin (Hb), hematocrit (HCT), erythrocyte sedimentation rate (ESR), platelet (PLT), mean corpuscular volume (MCV), mean corpuscular hemoglobin (MCH), mean corpuscular hemoglobin concentration (MCHC), and red blood cell distribution width (RDW) (Supplementary Table 1).

Parameters of biochemistry for redox status components including oxidative stress and antioxidant components were detected in the serum samples. All serum samples were assayed hydrogen peroxide (H_2_O_2_), malondialdehyde (MDA; as a method of thiobarbituric acid reactive substances-TBARS), total glutathione (GSH) contents, trolox equivalent antioxidant capacity (TEAC), catalase activities, superoxide dismutase (SOD) activities, cortisol, adrenaline, and serotonin, respectively. H_2_O_2_ in the serum level was analyzed by commercial kit as followed to manufacture’ manual (Amplex Red, Molecular Probes, Invitrogen Detection Technologies, Eugene, OR). Serum level of malondialdehyde (MDA), which is an end product of oxidation, was determined in serum levels of TBARS according to the previous method^15^. Briefly, 75 μL of serum sample was added to micro centrifuge tubes and transferred 3 μL of butylated hydroxytoluene in methanol. Then, 75 μL of 1 M phosphoric acid and equal volume of 2-thiobarbituric acid were added to same tube and mixed thoroughly with vortex. The mixed tubes incubated at 60 °C for 60 minutes. After incubation, 100 μL of each the reaction mixture was transferred to a microplate, and the absorbance was read using a spectrophotometer at both 535 and 572 nm to correct for baseline absorption (Versa Max. Molecular Device, Sunnyvale, CA). Tetramethoxypropane was used as a standard solution for calibration curve of quantification analysis. Total GSH contents in the serum level was determined according to a method described previously^16^. Briefly, either 50 μL of diluted serum (in 10 mM PBS, pH 7.2) or reduced GSH (as a standard) was combined with 80 μL DTNB/NADPH mixture (10 μL 4 mM DTNB and 70 μL 0.3 mM NADPH) in a 96-well plate. Then, 20 μL (0.06 U) of GSH-reductase solution was added to each well, and absorbance was determined by a plate reader at 405 nm. Antioxidant capacity was analyzed in serum via the TEAC method according to the previous described with slightly modified^17^. Each 90 μL aliquot of 10 mM PBS (pH 7.2), 50 ill myoglobin solution (18 gm.), 20 μL 3 mM ABTS solution, 20 μL diluted serum sample or various concentrations of Trolox (as a standard) were mixed into a 96-well microplate at 25 °C for 3 min. After then, 20 μL H_2_O_2_ was added to the each well and incubated for 5 min. The absorbance was measured using a plate reader at 600 nm. Serum levels of the SOD activity was measured using a commercial SOD assay kit (Dojindo Laboratories, Kumamoto, Japan), according to the manufacturer’s protocol, and bovine erythrocyte SOD (Sigma, St. Louis, MO, USA) was used as a calibration curve (0–50 U/mL). Serum catalase activities were assayed as described previously^18^. Catalase working buffer which is a mixture of PBS (250 mM, pH 7.2, for 15 μL), 12 mM methanol (for 15 μL), and 44 mM H_2_O_2_ (3 μL), which were total 33 μL were added to 96-well plates. Then, 30 μL of each sample/standard solution was added, and the reaction was proceeded for 10–20 min at RT. Reaction was stopped by adding 45 μL Purpald solution (22.8 mM Purpald in 2 N potassium hydroxide) and further incubated at RT for 20 min, followed by addition of 15 μL potassium periodate (65.2 mM in 0.5 N potassium hydrate). Absorbance was measured at 550 nm using a spectrophotometer.

### Determination of serum cortisol, adrenaline, serotonin levels

Serum cortisol and adrenaline, and serotonin levels were determined using commercially available of ELISA kits (cortisol, adrenaline, and serotonin, LDN GmbH & Co., KG, Nordhorn, Germany) respectively, according to the manufacturer’s protocol. Absorbance was measured using a spectrophotometer.

### Determination of serum tumor necrosis factor (TNF)-α and interferon (IFN)-γ levels

The serum level of TNF- α and IFN-γ were determined using a commercially available ELISA kit (Bio-Source, San Jose, CA). The procedures were followed using the manufacturer’s protocol. Each cytokine was determined at 450 nm and revised with 570 nm using a spectrophotometer (Soft Max 5.1).

### Statistical analysis

The average of each item for fatigue severity and the biochemical parameters of oxidative stress and antioxidants between the patients with CSD and healthy subjects were analyzed by Student’s t-test using SPSS (SPSS® 22. for Windows; SPSS, Inc., Chicago, IL, USA). A *p*-value < 0.05 was considered to indicate statistical significance.

## Results

### Physiological features of participants

To complete the present study, we gathered a total 199 participants and divided in to two groups which were as healthy participants (n = 152, male for 61 and female for 91) and patients with CSD (n = 47, male for 5 and female for 42) after diagnosed by a psychiatrist. The median height and body weight were 163 cm and 61.0 kg for healthy subjects, and 161 cm and 59.0 kg for the CSD subjects. The mean values of BMIs for each group were 23.1 for healthy subjects and 22.7 for the CSD subjects (Table 1). There were no statistical differences between each groups regarding the above contents.

**Table 1:** Physical features of participants.

Contents	Health	CSD
Number of subjects (%)	199 (100)	47 (100)
Male: Female	61 (31): 91 (69)	5 (11): 42 (89)
Median age	43 (18~65)	41 (19~65)
Male: Female	42 (19~65): 45 (20~65)	38 (24~52): 43 (19~65)
Heights (cm)	163 (154~184)	161 (152~178)
	173 (164~184): 159 (154~174)	172 (166~~178): 157 (152~168)
Body weights (kg)	61.0 (48.1~82.2)	59.0 (43.4~79.4)
	72.3 (55.7 ~ 82.2): 56.7 (48.1~71.8)	69.4 (61.3~79.4): 55.1 (43.4~63.7)
BMI (kg/m^2^)	23.1 (17.4~27.8)	22.7 (17.2~26.4)
	24.7 (19.1~27.8): 22.6 (17.4~27.5)	24.1 (21.1~26.2): 23.3 (17.2~26.4)

A total 199 of participants were enrolled to the present study and divided in to two groups the healthy and CSD. Before starting the present study, we estimated the base line of characters to all of participants. Data are expressed as Mean ± S.D. BMI; body mass index.

### Comparison of the questionnaire surveys

We assessed the DHI scores for analyzing self-perceived disability associated with the CSD. We determined the DHI which is contained three different areas including physical DHI, functional DHI, and emotional DHI, respectively. And all contents of above three were significantly increased as 3.4-, 3.5-, and 3.5-fold as compared with the healthy group (*p* < 0.05 for the Physical DHI and *p* < 0.001 for others, Table 2). Total DHI scores were 8.8 ± 2.1 in the healthy group, whereas CSD group was in 30.4 ± 5.6, respectively (*p* < 0.001, Table 2). The VAS score of the CSD group was approximately 2.4-fold higher than that of healthy group (*p* < 0.001, Table 2).

**Table 2:** Comparison of questionnaires surveys.

Measurements	Health	CSD
VAS	2.9 ± 1.7	7.0 ± 1.6***
Physical DHI	3.3 ± 1.8	11.1 ± 4.9*
Functional DHI	3.2 ± 0.2	11.2 ± 2.1***
Emotional DHI	2.3 ± 0.2	8.1 ± 1.1***
Total DHI score	8.8 ± 2.1	30.4 ± 5.6***

A total 199 of participants were enrolled to the present study and divided in to two groups the healthy and CSD. All participants answered to the questionnaire survey for measuring scores of DHI and VAS scores. Data are expressed as Mean ± S.D. *p < 0.05 and ***p < 0.001 for healthy group vs. CSD group. DHI; dizziness handicap inventory, VAS; visual analogue scale.

### Comparison of the serum levels of oxidative stress

**Figure 1:**
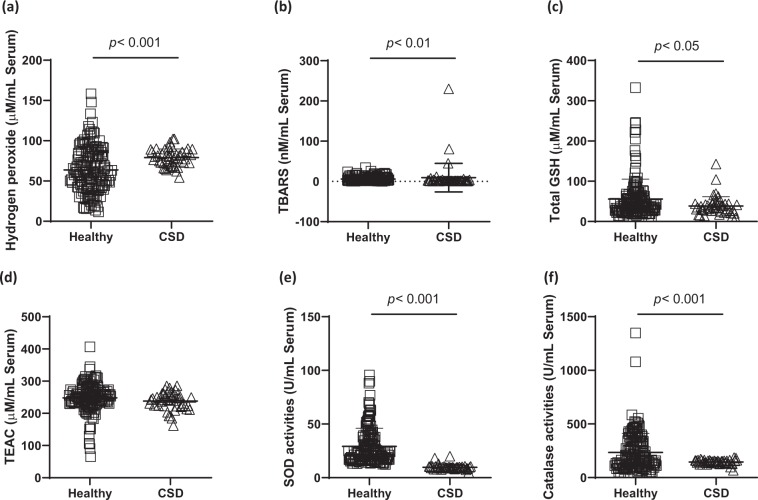
Serum levels of oxidative stress and endogenous antioxidant components. (**A**) Total three parameters of oxidative stress and four parameters of antioxidant components in the serum level were compared between 199 of healthy controls and 47 of CSD subjects. For oxidative stress markers, hydrogen peroxide (**a**), TBARS (**b**), total GSH (**c**), TEAC (**d**), SOD activities (**e**), and catalase activities (**f**). Data are expressed as the mean ± SD. *p < 0.05, **p < 0.01, and ***p < 0.001 for compared with the healthy group. CSD; chronic subjective dizziness, GSH; glutathione, SOD; superoxide dismutase, TBARS; thiobarbituric acid reactive substances, TEAC; Trolox equivalent antioxidant capacity.

To investigate the pathophysiological features the CSD, we firstly measured serum levels of oxidative stress markers including hydrogen peroxide and TBARS. In the CSD group, serum level of hydrogen peroxide in the CSD subjects was significantly higher, at 52.74 ± 7.09 nM, as compared to 42.46 ± 18.71 nM of the healthy group (*p* < 0.01, Fig. 1(a)). The final product of oxidative stress, lipid peroxidation in the serum levels, which was measured by TBARS in the CSD group were significantly altered, at 18.73 ± 70.86 nM as comparing to 5.94 ± 5.89 nM in the healthy group (*p* < 0.01, Fig. 1(c)).

### Comparison of the serum levels of antioxidant components

To verify the status of endogenous antioxidant components of the CSD patients, we analyzed non-enzymatic and enzymatic antioxidant components in the serum samples. Non-enzymatic, such as total GSH contents and total antioxidant capacities in the serum levels of the CSD group, the total GSH contents were significantly depleted at 37.72 ± 23.39 μM as comparing to 55.77 ± 49.50 uM of the healthy group (*p* < 0.05, Fig. 1(c)), but not abnormally altered in the serum levels of TEAC (253.15 ± 46.92 μM in the CSD group and 237.19 ± 26.72 μM in the healthy group, *p* > 0.05, Fig. 1(d)). Enzymatic antioxidant components including SOD and catalase activities were significantly decreased in the serum levels (at 29.21 ± 16.81 units in the CSD group and 9.73 ± 2.73 units in the healthy group for SOD; at 234.16 ± 178.12 units in the CSD group and 145.71 ± 20.06 units in the healthy group for catalase, *p* < 0.001 for both, Fig. 1(e,f)).

### Comparison of the serum levels of inflammatory cytokines

**Figure 2:**
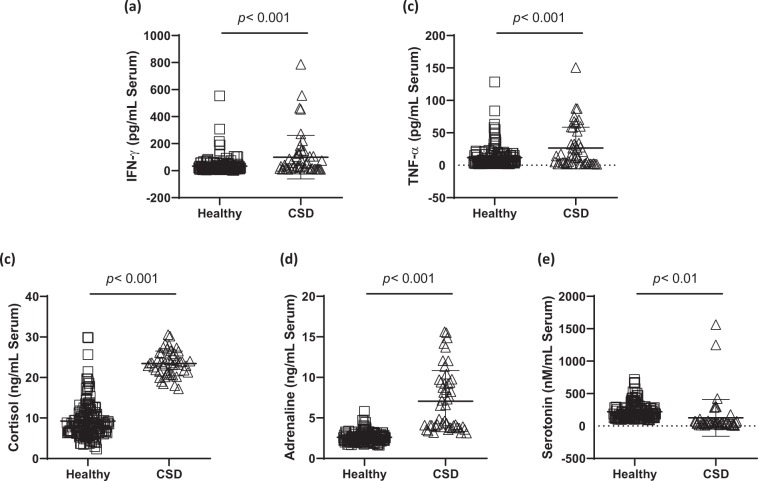
Serum levels of inflammation and stress related hormone. Inflammation related cytokines INF-γ (**a**) and TNF-α (**b**), and three kinds of emotional stress-related hormones including cortisol (**c**), adrenaline (**d**), and serotonin (**e**) in the serum level were measured, respectively. Data are expressed as the mean ± SD. **p < 0.01 and ***p < 0.001 for compared with the healthy group. CSD; chronic subjective dizziness, INF-γ; interferon-gamma, TNF-α; tumor necrosis factor-alpha.

Inflammatory related cytokines such as TNF-α and IFN-γ were measured in the present study. A representative pro-inflammatory cytokine, TNF-α in the serum levels of the CSD group were significantly increased as comparing to the healthy group (27.03 ± 32.35 pg/mL for the CSD group and 11.68 ± 16.24 pg/mL for the healthy group, *p* < 0.001, Fig. 2(a), respectively). As well known for the type II IFN, especially IFN- γ in the serum levels of the CSD was significantly elevated at 99.54 ± 160.92 pg/mL, as comparing to 33.54 ± 57.52 pg/mL of the healthy group (*p* < 0.001, Fig. 2(b)).

### Comparison of the serum levels of stress hormones

To observe relationship between the CSD and emotional, we further observed serum levels of cortisol, adrenaline, and serotonin in the present study. Serum cortisol levels of the CSD group, which were at 18.50 ± 9.16 ng/mL, were significantly higher than healthy groups which were at 234.63 ± 30.21 ng/mL (*p* < 0.001, Fig. 2(c)). The participants of the CSD in the serum adrenaline levels were at 570.58 ± 302.12 ng/mL, which were abnormally elevated with significance as comparing to the healthy group of adrenaline levels as 208.67 ± 51.22 ng/mL (*p* < 0.001, Fig. 2(d)). Serotonin in the serum levels of the CSD were at 127. 31 ± 287.47 nM, and this value was significantly lower than that of healthy group, at 219.02 ± 126.09 nM (*p* < 0.01, Fig. 2(e)).

### Relationships between endogenous redox components

**Figure 3:**
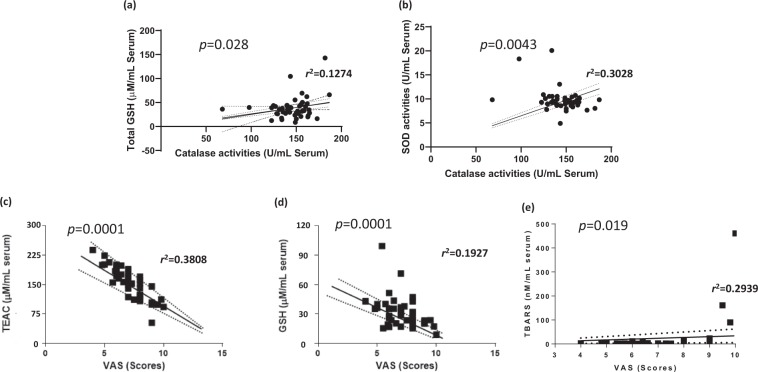
Regression analysis of variety parameters of the CSD patients. Pearson regression analysis between catalase activities and total GSH contents (**a**); catalase activities and SOD activities (**b**) were performed. Pearson regression analysis were also performed in the subjective severity of dizziness and endogenous redox status related parameters including total GSH contents (**c**), TEAC (**d**), and TBARS (**e**) in the serum levels of CSD.

To verify the pathological features of the CSD we further analyzed regression analysis for interactions between each of redox related parameters in the serum levels of the CSD participants. The endogenous antioxidant components between total GSH content catalase activities were significantly interacts each other (Fig. 3(a), *p* < 0.01). The two of enzymatic antioxidant components, catalase and SOD are also well correlated in the CSD patients of their activities of the serum levels (Fig. 3(b), *p* < 0.05). The subjective measurement of severity of dizziness are well reversely correlated to total GSH and TEAC (Fig. 3(c,d), *p* < 0.01 and *p* < 0.001 for TEAC), however showed a dependent manner of TBAR values (Fig. 3(e), *p* < 0.05), respectively.

## Discussion

Previous studies have tried to reveal for studying an accurate pathophysiological reason of this disease globally. These efforts accomplish to validate the primary symptoms of the CSD; however, the CSD generally are generated by interactions between neuro-otologic and behavioral elements, therefore it is difficult for clinicians to accurately recognize and categorize of patients with the CSD. Therefore, most of patients with the CSD is diagnosed by its subjective symptoms. Because this disorder arises from complexity, the therapeutic accesses are also carefully approached relying on the symptoms. In addition, the pathophysiological status of the CSD still remains unclearly.

In the present study, due to above issues, we aimed to observe the possible parameters of the CSD in an endogenous parameter of human body. The first hypothesis in the present study was to prove the endogenous redox system imbalance would be related to the CSD or not. Accumulated studies evidenced that the CSD is mainly triggered neuro-otologic and psychiatric disorders^13,19,20^. Furthermore, recent studies reported that the above pathological alterations are deeply related to the oxidative stress in clinical practice and animal based *in vivo* experiment^21–23^.

To verify the oxidative stress status in the patients with the CSD, we analyzed the redox system in the serum levels. First finding in the present study and as our expectation, the serum levels of H_2_O_2_ and TBARS levels were significantly higher 1.24- and 3.15-fold than that of the healthy groups (*p* < 0.001 and *p* < 0.01, Fig. 1(a,b)). Accumulated evidences well reported that oxidative stress, under the condition of excessive ROS generation extremely deplete endogenous antioxidant components, then leads to imbalance redox system^24,25^. These abnormal conditions is well known for wide range of pathophysiological progressions, such as inflammation, cardio vascular disorders, cancers, aging, and psychiatric disorders^26–28^. Biologic organisms generate energy for maintain its own life from oxygen reduction are forced to produce ROS. Thus they have evolved antioxidant defense mechanisms by endogenous enzymatic antioxidants and non-enzyme derived antioxidant molecules^29^. Data from the current study, total GSH contents and both SOD and catalase activities were significantly different in the healthy control group (*p* < 0.05 or *p* < 0.001, Fig. 1(c,f,g)), but not significant alterations of TEAC (Fig. 1(d)). Inflammatory cytokines such as IFN-γ and TNF-α serum levels in the CSD patients were significantly increased than that of the healthy group (*p* < 0.001, Fig. 2(a,b)). It is well known that the oxidative stress is deeply associated with the inflammation. IFN-γ can induce oxidation by inhibition of antioxidant effects^30^, and TNF-α is triggered by oxidative stress or released by response of oxidative stress^31^.

Additionally, the symptoms of the CSD is also linked to the emotional behaviors. Thus we further examined emotional stress related hormones secretion. Stress-related hormones are generally provoked by response to psychological stress-mediated condition which could be also affected by circadian rhythms^32^. To avoid this condition that circadian rhythms hinder stress hormone secretions, we gathered whole blood sample exact time point between 2:00 to 3:00 pm though all subjects. According to our results, we manifestly observed the significant elevations of cortisol, adrenaline, and decreases of serotonin as comparing to the healthy group (*p* < 0.001 for *p* < 0.01, Fig. 2(c–e)). Our hypothesis is that the CSD may lead to provoke abnormal emotional stress status, and this event provokes alterations of stress related hormone releases. It is well known that the cortisol is abnormally released from adrenal cortex as a response of stress^33^, whereas a representative sympathetic hormones, epinephrine, is well known to be released actively into the blood under the both psychological or physical stress via activation of sympathetic nerve^34^. Serotonin, contrary to the above, beneficially works to psychological disorders, such as depression and anxiety^35^. Therefore, our results well supplemented that emotional dysfunction related stress hormone releases were abnormally altered in the CSD group.

Interestingly, we observed that the endogenous antioxidant components in the serum levels of the CSD subjects are deeply linked to each other, especially catalase activities with total GSH contents, and catalase activities with SOD activities (Fig. 3(a,b)). In addition, subjective severity of dizziness which was measured by VAS score were reversely correlated to the total GSH contents as well as TEAC in the serum levels (Fig. 3(c,d)), however this phenomenon showed dependent manners with TBARS levels (Fig. 3(e)). These findings well evidenced that even in the CSD disorders, the experimental based parameters will be objective diagnose markers in the medical field.

Excessive oxidative stress also contributes to alteration of membrane integrity and fragility of RBCs^36,37^. Thus, we further analyzed hematological test analysis especially focusing on the RBC and its related parameters. Our data exhibit hemoglobin and MCHC levels were significantly altered in the CSD group as compared with the health group, but other parameters were not altered between groups (*p* < 0.05 for hemoglobin and *p* < 0.01 for MCHC, respectively Supplementary Table 1). Although we observed the statistically significant differences between groups, these levels were including normal ranges in each group. Therefore, in the current study, there were no crucial clues to explain the relationships of oxidative stress condition and RBCs or their related parameters.

Unfortunately, the exact cause of CSD is still being worked out; however, it is highly doubted due to inabilities of readjustment of brain after the vestibular system damage^12,38^. Although many of medical inspections are relying on the diagnosis criteria of the CSD based on the subjective perception by questionnaire survey, it sounds urgently to figure out and understand convincing contributors to accomplish to the CSD. Additionally, many of studies have made efforts to try to develop various therapeutics to treat the CSD in clinical practice, still lack of accumulated knowledges of efficient treatments.

In the present study, we explored the possible parameters which can mediate the CSD via enhancement of oxidative stress and deteriorations of antioxidant components. Furthermore, these abnormal imbalances also lead to alter stress hormone system as well as inflammatory response in the CSD patients. From our data, we firstly demonstrated that oxidative stress is intensively involved in the pathology of CSD compared to a healthy population. These data will be helpful us to further explore the pathophysiology of CSD.

## Supplementary information

Supplementary Information.
