# Supplementary material for: Biomarkers of Oxidative Stress and Endogenous Antioxidants for Patients with Chronic Subjective Dizziness
Source: Sci Rep. 2020 Jan 30;10:1478. doi: 10.1038/s41598-020-58218-w (PMC6992639; doi:10.1038/s41598-020-58218-w)
Supplement: Supplementary file 1 — Supplementary Information. [file 41598_2020_58218_MOESM1_ESM.pdf]

Supplementary information

Biomarkers of Oxidative Stress and Endogenous Antioxidants for Patients with Chronic Subjective Dizziness

Zhigang Fang[1,2,3], Keer Huang[2], Chang-Hyun Gil[4], Jin-Woo Jeong[5],

Ho-Ryong Yoo [6, a, #], and Hyeong-Geug Kim[3, a,#]

[1]*Guangzhou University of Chinese Medicine, Guangzhou 510405, PR China*

[2]*The First Affiliated Hospital of Guangzhou University of Chinese Medicine, Guangzhou 510405, PR China*

[3]*Department of Biochemistry and Molecular Biology, Indiana University School of Medicine, Indianapolis, IN 46202*

[4]*Division of Vascular Surgery, Department of Surgery, Indiana University School of Medicine, Indianapolis, IN 46202*

[5]*Researcher, Animal & Plant Utilization Team, Nakdonggang National Institute of Biological Resources, 137, Donam 2-gil, Sangju-si, Gyeongsangbuk-do 37242, Korea*

[6]*Department of Neurology Disorders, Dunsan Oriental Hospital of Daejeon University, 75, Daedeok-daero 176, Seo-gu, Daejeon, 35253, South Korea.*

Material and methods

Laboratory tests. Whole blood of all participants were isolated from forearm needle and Vacutainer™ tubes by a trained phlebotomist. Hematological analysis were performed in laboratories at Oriental Hospital of Daejeon University using standardized procedures.

Supplementary Table 1. Hematological Analysis of red blood cell populations

Measurements	Health		CSD	
	Mean ± S.D.	Reference range	Mean ± S.D.	Reference range
RBC (10^6/mL)	4.467 ± 0.46	3.72 ~ 6.29	4.367 ± 0.39	3.51 ~ 5.24
Hemoglobin (g/dL)	13.66 ± 1.66	7.5 ~ 17.1	13.10 ± 1.58$^{\#}$	8.0 ~ 16.7
Hematocrit (%)	39.89 ± 4.25	24.8 ~ 50.1	39.25 ± 4.16	26.8 ~ 49.1
ESR (mm/hr)	11.03 ± 8.35	1.0 ~ 48.0	11.85 ± 8.12	2.0 ~ 38.0
Platelet (10^4/μL)	24.53 ± 5.78	14.5 ~ 47.3	24.01 ± 4.64	16.4 ~ 36.0
MCV (fl)	88.73 ± 9.89	61.5 ~ 100.0	90.02 ± 6.81	66.0 ~ 102.0
MCH (pg)	30.49 ± 2.91	16.8 ~ 34.7	29.99 ± 2.76	19.8 ~ 34.8
MCHC (%)	34.85 ± 5.91	28.6 ~ 55.7	33.28 ± 0.84$^{\#\#}$	30.0 ~ 34.7
RDW (%)	12.88 ± 2.01	11.5 ~ 32.6	12.55 ± 1.51	10.6 ~ 18.5

A total 199 of participants were enrolled to the present study and divided in to two groups the healthy and CSD. Whole blood samples were collected from arm vein of all participants. Data are expressed as Mean ± S.D. RBC, red blood cells; ESR, erythrocyte sedimentation rate; MCV, mean corpuscular volume; MCH, mean corpuscular hemoglobin; MCHC, mean corpuscular hemoglobin concentration; RDW, red cell distribution width. $P^{*} < 0.05$ and $P^{**} < 0.05$ for healthy group *vs.* CSD group
